# Global COVID-19 Vaccine Inequity: Failures in the First Year of Distribution and Potential Solutions for the Future

**DOI:** 10.3389/fpubh.2022.821117

**Published:** 2022-03-07

**Authors:** Victoria Pilkington, Sarai Mirjam Keestra, Andrew Hill

**Affiliations:** ^1^Oxford University Clinical Academic Graduate School, University of Oxford, Oxford, United Kingdom; ^2^MetaVirology Ltd., London, United Kingdom; ^3^Epidemiology and Data Science, Amsterdam University Medical Centre, University of Amsterdam, Amsterdam, Netherlands; ^4^Department for Translational Medicine, Liverpool University, Liverpool, United Kingdom

**Keywords:** manufacturing—R&D interface, pricing, vaccines, COVID-19, inequality

## Abstract

Within the first year of distribution of vaccines against COVID-19, high-income countries (HICs) have achieved vaccination rates of 75-80%, whilst low-income countries (LICs) vaccinated <10%. This disparity in access has been one of the greatest failures of international cooperation during the SARS-CoV-2 pandemic. Global COVID-19 vaccine inequity affects us all, with ongoing risk of new variants emerging until global herd immunity is strengthened. The current model of global vaccine distribution is based on financial competition for limited vaccine supplies, resulting in HICs getting first access to vaccines, with LICs being forced to rely on voluntary donations through schemes like COVAX. Pharmaceutical companies own the intellectual property (IP) rights for COVID-19 vaccines, allowing them to control manufacturing, distribution, and pricing. However, the pharmaceutical industry did not develop these vaccines alone, with billions of dollars of public funding being instrumental in their discovery and development. Solutions to enable global equitable access already exist. The next step in scale up of manufacture and distribution worldwide is equitable knowledge sharing and technology transfer. The World Health Organization centralized technology transfer hub would facilitate international cooperation. Investments made into developing this infrastructure benefit the COVID-19 response whilst promoting future pandemic preparedness. Whilst globally there is majority support for waivers of IP to facilitate this next step, key opponents blocking this move include the UK and other European countries which host large domestic pharmaceutical industries. A nationalistic approach is not effective during a global pandemic. International cooperation is essential to achieve global goals against COVID-19.

## Summary Box

What is already known:

- Within the first year of distribution of vaccines against COVID-19, high-income countries (HICs) have achieved vaccination rates of 75-80%, whilst low-income countries (LICs) have vaccinated <10%.
- The pharmaceutical industry did not develop these vaccines alone, with billions of dollars of public funding being instrumental in their discovery and development. However, private companies who hold IP currently control manufacturing, distribution, and pricing.
- The current model of global vaccine distribution is based on financial competition for limited vaccine supplies, resulting in HICs getting first access to vaccines, with LICs being forced to rely on voluntary donations through schemes like COVAX.

What are the recommendations for policy:

- Solutions exist, such as using a WHO centralized technology transfer hub to facilitate worldwide knowledge sharing and scale up manufacture. These are only possibly if there is increased global cooperation and support for a waiver on intellectual property for COVID-19 technologies.

## Inequities in Vaccine Access

The rapid development of a new vaccine against SARS-CoV-2 has been rightly celebrated as a breakthrough in the response to the pandemic, reducing hospitalization and death from COVID-19. Over 9 billion doses were administered by the end of 2021 (1). In contrast, the ongoing inequality in vaccine distribution remains one of the greatest failures of international cooperation during the COVID-19 pandemic. Whilst higher income countries (HICs) have been able to move quickly and vaccinate most of their populations, populations in other areas of the world continue to have limited access to vaccines against COVID-19 (2).

**Figure 1:**
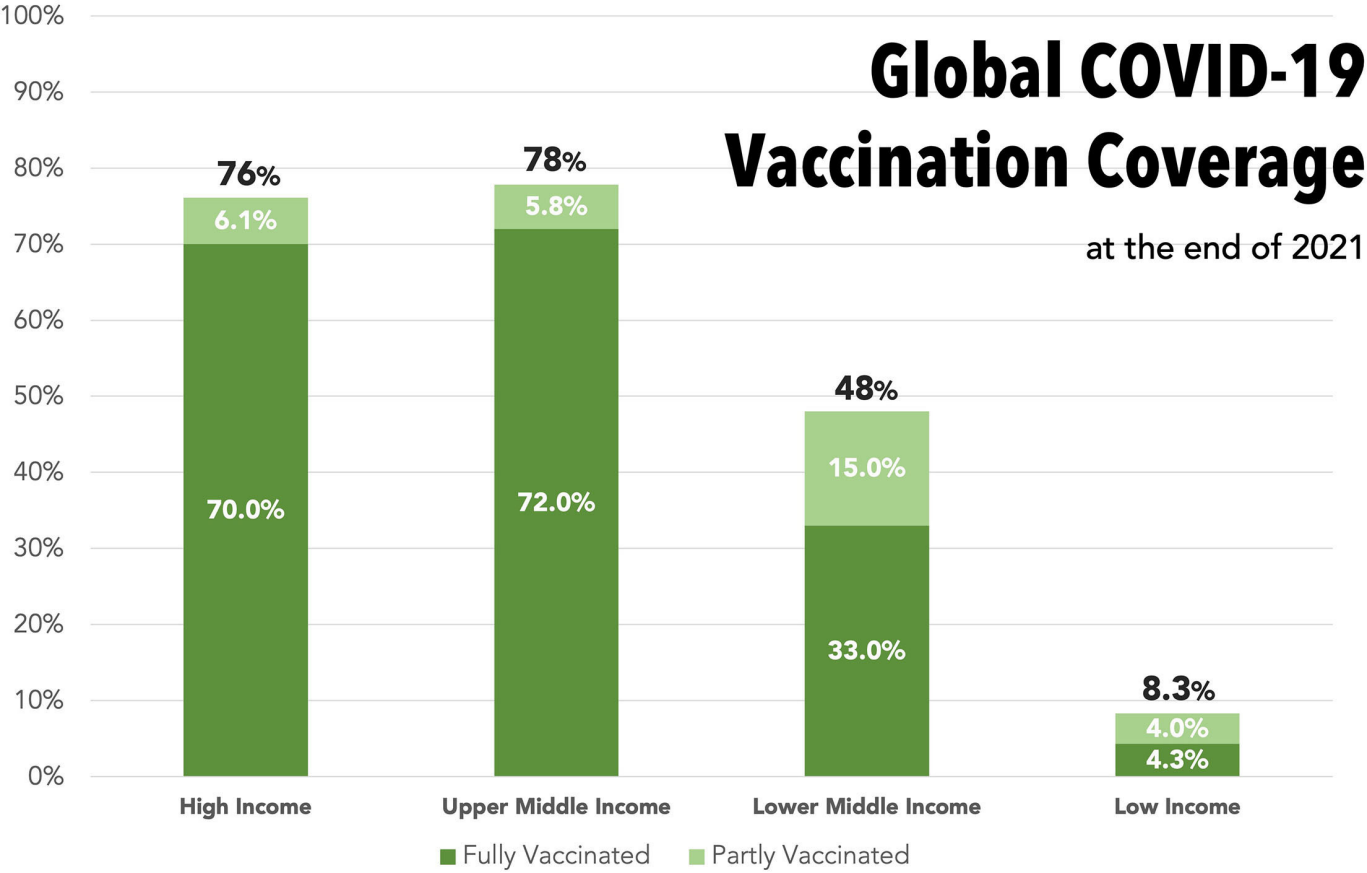
Bar graph displaying the proportions of the overall population who had received part (1st vaccine) or completed (2nd vaccine) a COVID-19 vaccination course by the end of 2021, stratified into World Trade Organization income categories. Dark green, completed course; Light green, partially vaccinated. Total proportion who have received any vaccine is stated at top of each bar. Data from Our World in Data—timepoint 31/12/21 (1).

Global disparities in vaccine access are stark. The first COVID-19 vaccines received emergency regulatory approval in December 2020 (3). Within the first year of distribution, HICs have been able to hit their targets and vaccinate 75-80% of their populations. During the same period, low-income countries (LICs) vaccinated <10% of their populations due to inequalities in vaccine access (1) (Figure 1).

High-income countries across Europe and North America have hoarded many more vaccine doses than they need (4), tying up the global vaccine supply. There have even been reports of widespread vaccine wastage in HICs, with up to 15 million doses thrown away in the United States alone between March and September 2021 (5). The WHO Director General reported that, by November 2021, more than 80% of the world's vaccines had gone to G20 countries, whereas LICs had received just 0.6% of all vaccines (6). The pressure on global supply chains looks set to continue, with ongoing need for booster doses. International institutions, such as the World Health Organization (WHO) and global health advocates such as Médecins Sans Frontières (MSF), have called for a pause to booster programs to support a global effort to vaccinate at least 10% of every country's population (7), but booster vaccination programs across HICs continue.

It has been estimated that 60-70% of the world needs to be vaccinated to achieve “global immunity” (8). Mathematical modeling has shown that the optimal vaccine allocation strategy, to slow international spread and avert unnecessary deaths, would be to distribute vaccines according to country population size, in combination with prioritized roll out to high-risk groups and key transmitters (9). In fact, mathematical modeling suggests that when HICs preferentially obtain the majority of the available vaccine doses at the expense of lower income countries (LICs), this could result in 900 additional deaths per million of the world population, equating to millions of excess deaths globally (9). Médecins Sans Frontiers (MSF) have estimated that, if available vaccine doses were steadily redistributed, nearly one million deaths would be averted by mid-2022 compared to the current scenarios (2).

Inequities in COVID-19 vaccine access do not just impact those who are unable to get immunized, they affect all of us. Millions of new cases are reported every day worldwide, bringing with them the possible emergence of more infectious variants, as seen with Omicron. The continuing pandemic also comes at a huge cost to the global economy, estimated to be at least $16 trillion so far (10). Indeed, the Eurasia Group has estimated that the economic benefits, for 10 key HIC economies alone, are as much as $466 billion between 2020 and 2025 if we achieve a global equitable vaccine solution (11). Additionally, there is an ongoing toll on mental health (12), loss of livelihoods, and splitting of families, all contributing to an undeniable and unquantifiable cost to quality of life around the world.

## The Role of High-Income Countries

COVID-19 Vaccines Global Access Scheme (COVAX) has been the leading international scheme to facilitate global COVID-19 vaccine distribution during 2021. It acts as a centralized vaccine buyer's club, either purchasing vaccines directly from pharmaceutical manufacturers or receiving donations from HICs. Sixty seven low-income countries (LICs) rely on COVAX for their COVID-19 vaccine supplies (13).

Governments of HICs have proudly hailed their pledges to donate hundreds of millions of vaccine doses to COVAX (14) as instrumental in global vaccination efforts. However, this is just a fraction of the billions of doses needed to vaccinate the worldwide population. Even in the most optimistic scenarios, COVAX only ever hoped to support vaccination of 20% of its target populations in 2021, many times less what is needed to achieve global immunity (15).

The reality was even less successful. COVAX repeatedly failed to hit key targets. Projections were scaled back and a 25% reduction in anticipated volumes of vaccine available compared to initial forecasts in early 2021 (16). African countries received only 18.2 million of the 66 million doses they had expected through COVAX in the first half of 2021 (17), and most did not reach even 10% vaccination by the end of 2021 (1, 18).

The current model of global vaccine distribution has been based on financial competition for limited vaccine supplies. This has resulted in HICs getting prioritized access by purchasing vaccines through bilateral deals with pharmaceutical companies, tying up global vaccine supplies (19). Low and middle-income countries (LMICs), and even COVAX itself, have therefore struggled to purchase any vaccines and instead have been forced to wait and rely on voluntary donations, many of which are unusable after not being donated until already close to expiry (20).

The COVAX model focuses on the redistribution of existing vaccine supplies, but this has proven to be insufficient. Pressure on global supplies will continue as booster dose programs are rolled out, further diverting much needed doses from those countries struggling to complete even initial vaccine courses. There is no doubt that production needs to be scaled up significantly to meet growing global demand.

## The Role of the Pharmaceutical Industry

A variety of vaccines have now been granted WHO approval for clinical use against COVID-19, including non-replicating viral vector, inactivated, and mRNA vaccines (21). Intellectual property (IP) rights allow the pharmaceutical companies involved in developing these vaccines to control the manufacture of vaccine supplies and therefore control pricing (22).

However, these industry developers were not the sole providers of the funding for the research and development (R&D) underpinning the new COVID-19 vaccines (23). In the United States alone, the Biomedical Advanced Research and Development Authority (BARDA) has awarded more than $10 billion of public funds to the development of COVID-19 vaccines (24). The extent of the public funding contribution, particularly to the Moderna mRNA vaccine, could justify the government invoking step-in rights regarding the IP through the Bayh-Dole Act 1980 (25). In the UK, public and charitable funders accelerated the development of the AstraZeneca vaccine (26, 27) and the European Union has spent an estimated €93 billion on public sector investment in COVID-19 vaccines and therapeutics R&D (27).

Public investment was instrumental in accelerating COVID-19 vaccine discovery, with technologies often being based on decades of academic research (27, 28). Public investment also supported vaccine development through clinical development stages, with the use of government facilities, research grants, and by accelerating regulatory approval (29). Public investment was then used to underwrite the risk of production costs, with advance purchase schemes (30). A network analysis of patent holders does not identify any single inventor of the health technologies that made the COVID-19 vaccines possible, but instead describes a complex web of licensing agreements held between entities who jointly contributed (31). This is because knowledge generation is most often a collective process, spanning decades and involving multiple actors.

Despite this public investment, the control of IP for COVID-19 vaccines remains with private industry. This means that vaccine supplies can only be manufactured when licensed and with the technology transfer overseen by these companies. This, in turn, results in prices set based on monopolistic access to a market, leading to prices that are well over the estimated costs of production.

**Figure 2:**
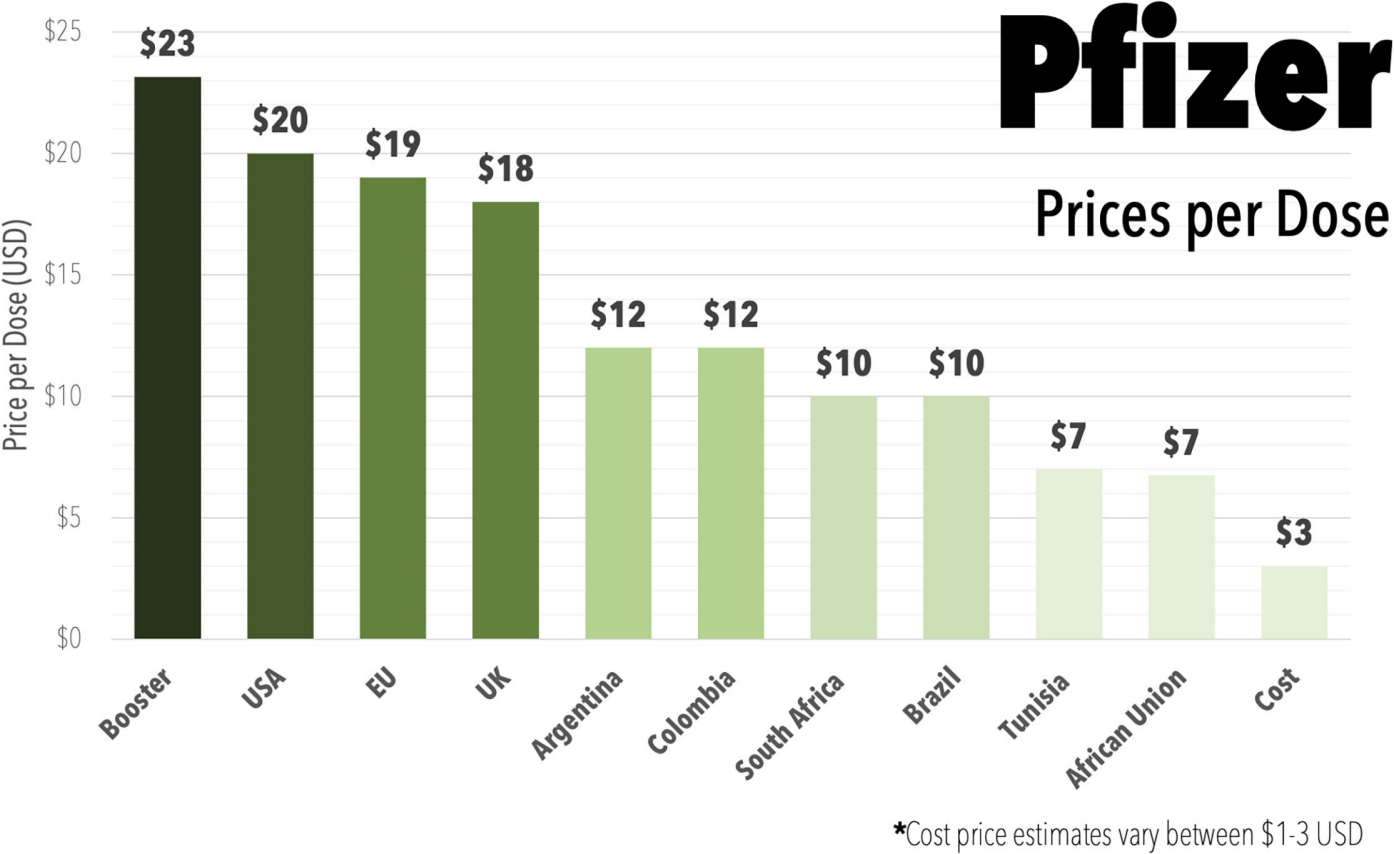
Bar graph displaying the range of prices reported to be paid in different countries or areas of the world for the Pfizer mRNA COVID-19 vaccine (Comirnaty) during 2021, including reported higher prices being charged for booster doses and including estimate on the cost of production. Data Oxfam Coronavirus Dashboard (32). Cost price estimate from (33).

Governments negotiate a variety of different prices through bilateral deals shrouded in secrecy (Figure 2), with the mRNA vaccines by Pfizer and Moderna being the most expensive (32). However, the estimates of manufacturing costs for these mRNA vaccines are as low as $1-3 USD per dose (33) (Figure 2). The lack of transparency in pricing works to the advantage of the pharmaceutical industry, rather than the international community. For example, the UK is estimated to have overpaid $1.8 billion over the cost of production for the Pfizer and Moderna vaccines, the United States by $17.4 billion, and the EU by €31 billion (34).

The profits being made from COVID-19 vaccines flow back to private pharmaceutical companies and their shareholders. Sales of AstraZeneca's vaccine has brought in $1.2 bn (£900 m) in the first half of 2021, with sales tripling in the second quarter from the first (35). After their pledge to sell the vaccine “at cost” during the pandemic, they declared the pandemic over in November 2021, prior to the Omicron wave (36). Moderna projected $19.2 billion in COVID vaccine sales in its 2021 second quarter report, with Moderna shares increasing almost 700% this year (37, 38). Pfizer projected sales of $26 billion for 2021 alone, before increasing this projection to $33.5 billion dollars (37, 39).

Private companies did not develop the COVID-19 vaccines alone. Instead, these discoveries were made based on collective knowledge generated by public investment into R&D, and public funding was instrumental in this process. Therefore, private companies should not control decisions on manufacturing, distribution and profit alone. Now that the vaccine technology has been developed and approved, the next crucial step is knowledge sharing and technology transfer globally. It is important that intellectual property, including manufacturing knowhow, is shared on an equitable basis to facilitate sustainable scale up of manufacture and distribution worldwide.

## Potential Solutions

Proposed solutions to improve global vaccine distribution have included compulsory licensing, which is complex and limited until full patents are granted (40, 41), and expanding the COVAX initiative which has already proved to be insufficient on its own. A leading proposal for redistributing control of essential vaccine IP, and thereby the power over up-scaling manufacture, is the TRIPS waiver. This was proposed by India and South Africa at the World Trade Organization (WTO) in October 2020 (42). The Agreement on Trade-Related Aspects of Intellectual Property Rights (TRIPS agreement) is an international legal agreement between WTO member states. It protects against the infringement on intellectual property, for example by preventing the generic manufacture of patented medicines (43). The proposed TRIPS waiver would be an agreement between WTO member states not to impose these trade sanctions for the manufacturing and sale of patented COVID-19 health technologies, to facilitate global sharing of IP.

Although the TRIPS waiver is supported by the great majority of WTO countries, some key HICs have opposed it and unanimous support is necessary to pass the motion (44). The United States have offered support with the key limitation that the waiver should apply to vaccines only, not COVID-19 therapeutics and diagnostics (45) and they also specific that the sharing of technologies would be on a voluntary basis only. Other countries which host large domestic pharmaceutic industries, including the UK and Germany, have been key opponents of the waiver (46). These countries may oppose the waiver on an economic basis. Estimates have suggested that BioNTech, the biotechnology company behind Pfizer's mRNA vaccine, alone could boost Germany's GDP by 0.5% in 2021 (47). However, a nationalistic approach based on economic interests is not effective in the global fight against a pandemic disease which knows no borders.

Opponents of the TRIPS waiver cite concerns regarding the importance of IP in incentivising innovation (46). However, during the special circumstances of the pandemic innovation was highly incentivised with public funding provided up front, de-risking R&D. The TRIPS waiver would still allow for compensation to the companies who hold patent rights, but would redistribute control of the production of the technologies and allow for greater manufacturing capacity (42). This would likely reduce industry profits as greater supply and biosimilar competition would result in lower prices (48). Although pharmaceutical companies have undoubtedly aided the global COVID pandemic response, the welfare of the global community must be put before private profits.

Relinquishing control of IP is an important first step toward global vaccine access. The next step would be equitable technology transfer and knowledge sharing from pharmaceutical companies to biosimilar manufacturers. Existing bilateral technology transfer already takes place (49) between internal pharmaceutical manufacturing sites and designated outsourced facilities (50). Manufacturing sites around the world have already been identified as candidates in scaling up biosimilar vaccine production (51). India alone has the pre-existing infrastructure in place to manufacture upwards of 3 billion vaccines doses a year and a long-standing track record of cost-effective biosimilar vaccine manufacture on a large scale (52) and a commitment to support equitable vaccine distribution and provide vaccine assistance to LICs (53). However, patent holders rarely voluntarily agree to share manufacturing knowhow externally to facilitate this. Only forcing a release of IP will facilitate this knowledge sharing.

**Figure 3:**
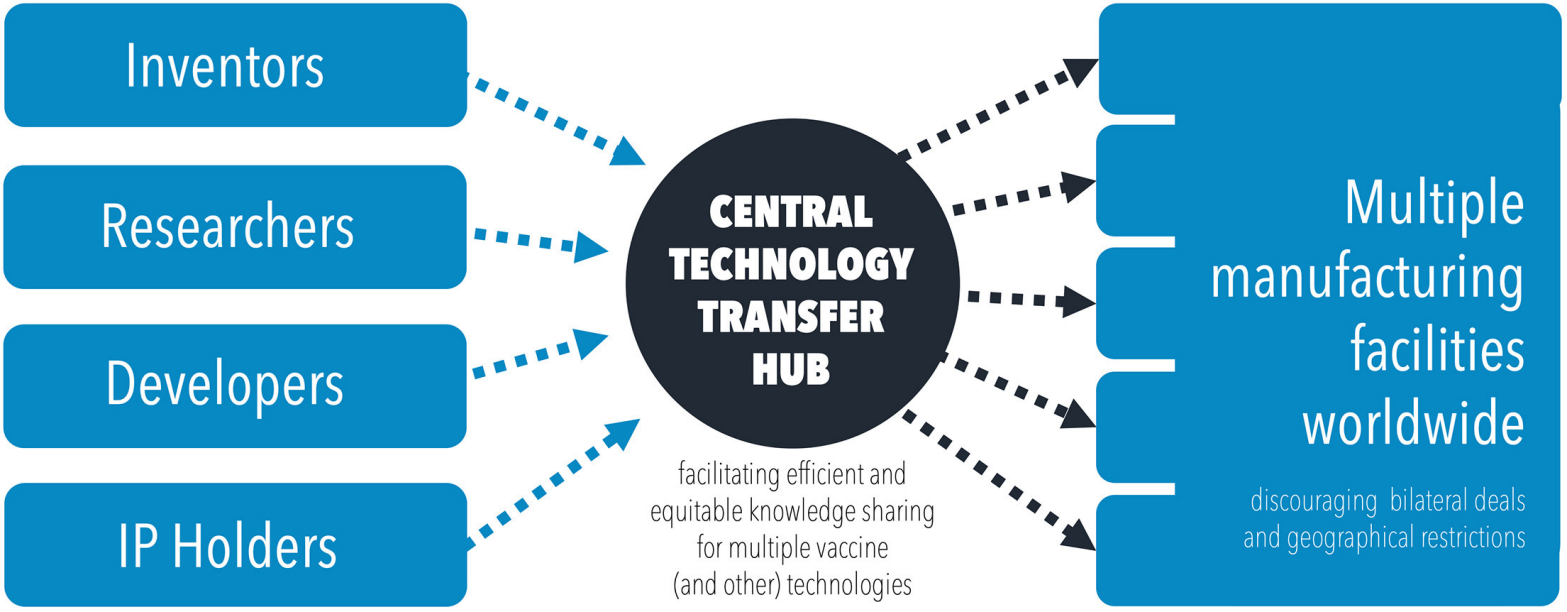
Diagram illustrating the flow of knowledge and technology transfer through a centralized technology transfer hub, to disseminate information and scale up manufacturing efforts.

With IP limitations removed, a more collaborative, centralized, technology transfer hub would be possible (Figure 3), promoting global vaccine diplomacy (53). The World Health Organization have already proposed that they will facilitate such a hub, specifically for mRNA technology transfer (54). Similar efforts should be made to centralize and share the technical knowhow for other vaccine technologies, beyond mRNA vaccines. A centralised model has the added benefits of improving coordination, providing support, allowing for global surveillance of vaccine efforts and improving quality control across manufacturing sites. All of this acts to promote global cooperation, leading to sustainable and equitable global vaccine security. The global scale up of production of mRNA vaccines alone has been estimated to have the potential to avert 1.3 million COVID-19 deaths by improving vaccination rates in LIC/LMICs (55).

Structural limitations remain, and the need for improved infrastructure, training, equipment and materials in resource limited settings (41). Any mechanisms for knowledge sharing and technology transfer would need to be accompanied by planning and sustainable funding in this area as well. Investments made into developing this infrastructure and global technology transfer mechanisms would not only benefit the COVID-19 response, but would also promote future pandemic preparedness (56). If the model of a centralized international technology transfer hub proofs effective for sharing knowledge on vaccine manufacturing, the same methodology should be applied to the sharing of other key COVID-19 health technologies such as diagnostics and therapeutics.

## Conclusions

The World Bank, the IMF, the WTO, and the WHO have set a goal of 70% vaccination worldwide by mid-2022. It has become increasingly clear that the current situation, with few actors controlling the vaccine manufacture and supply and a reliance on voluntary donations *via* COVAX, is not an equitable, sustainable or efficient system and will fail to achieve this target. Rather than being forced to rely on voluntary donations, the governments of LMICs are asking for access to purchase COVID-19 vaccines at an affordable price, close to cost of production, and the opportunity to manufacture supplies to ensure future pandemic preparedness. Increasing international cooperation around technology transfer and the sharing of essential manufacturing knowhow is essential.

Private companies restrict the opportunity to end the pandemic sooner by not participating in international technology transfer efforts. Meanwhile, the same countries which host these private companies perpetuate this situation by opposing the TRIPS waiver at the WTO, buying up the existing global supplies at exorbitant prices. This artificial global shortage and the resulting continuation of the COVID-19 pandemic, with all of the associated economic impacts and entirely avoidable deaths, can therefore be seen as a form of structural violence. Whilst these issues are debated, thousands continue to die worldwide every day and millions more face lasting illness and morbidity induced by COVID-19 infection. At least 5.6 million people have already died from COVID-19 at the time of writing (1). Although vaccinations alone will not resolve the COVID-19 pandemic, global equitable access to these key health technologies is an important part of the worldwide effort to fight this disease. International cooperation is needed now to achieve the global COVID-19 vaccination goals that were not achieved in 2021, in the year 2022.

## Data Availability Statement

The original contributions presented in the study are included in the article/supplementary material, further inquiries can be directed to the corresponding author/s.

## Author Contributions

VP and SK prepared the manuscript. VP designed the figures. AH supervised and reviewed the manuscript. All authors approved the final manuscript.

## Funding

This research was funded by research grants from the International Treatment Preparedness Coalition and the Make Medicines Affordable Campaign.

## Conflict of Interest

VP has previously worked with Universities Allied for Essential Medicines Europe on voluntary basis, but has no other competitions of interest to declare. SK is an active voluntary member of Universities Allied for Essential Medicines Europe. However, views expressed in this paper are her own and are not necessarily shared with the organizations the authors are affiliated with. The remaining author declares that the research was conducted in the absence of any commercial or financial relationships that could be construed as a potential conflict of interest.

## Publisher's Note

All claims expressed in this article are solely those of the authors and do not necessarily represent those of their affiliated organizations, or those of the publisher, the editors and the reviewers. Any product that may be evaluated in this article, or claim that may be made by its manufacturer, is not guaranteed or endorsed by the publisher.
